# Supplementary material for: Inflammatory factors and risk of lung adenocarcinoma: a Mendelian randomization study mediated by blood metabolites
Source: Front Endocrinol (Lausanne). 2024 Aug 27;15:1446863. doi: 10.3389/fendo.2024.1446863 (PMC11384989; doi:10.3389/fendo.2024.1446863)
Supplement: Supplementary file 1 [file DataSheet1.zip › supplementary files/S2/GCST90200392/sensitivity-analysis.pdf]

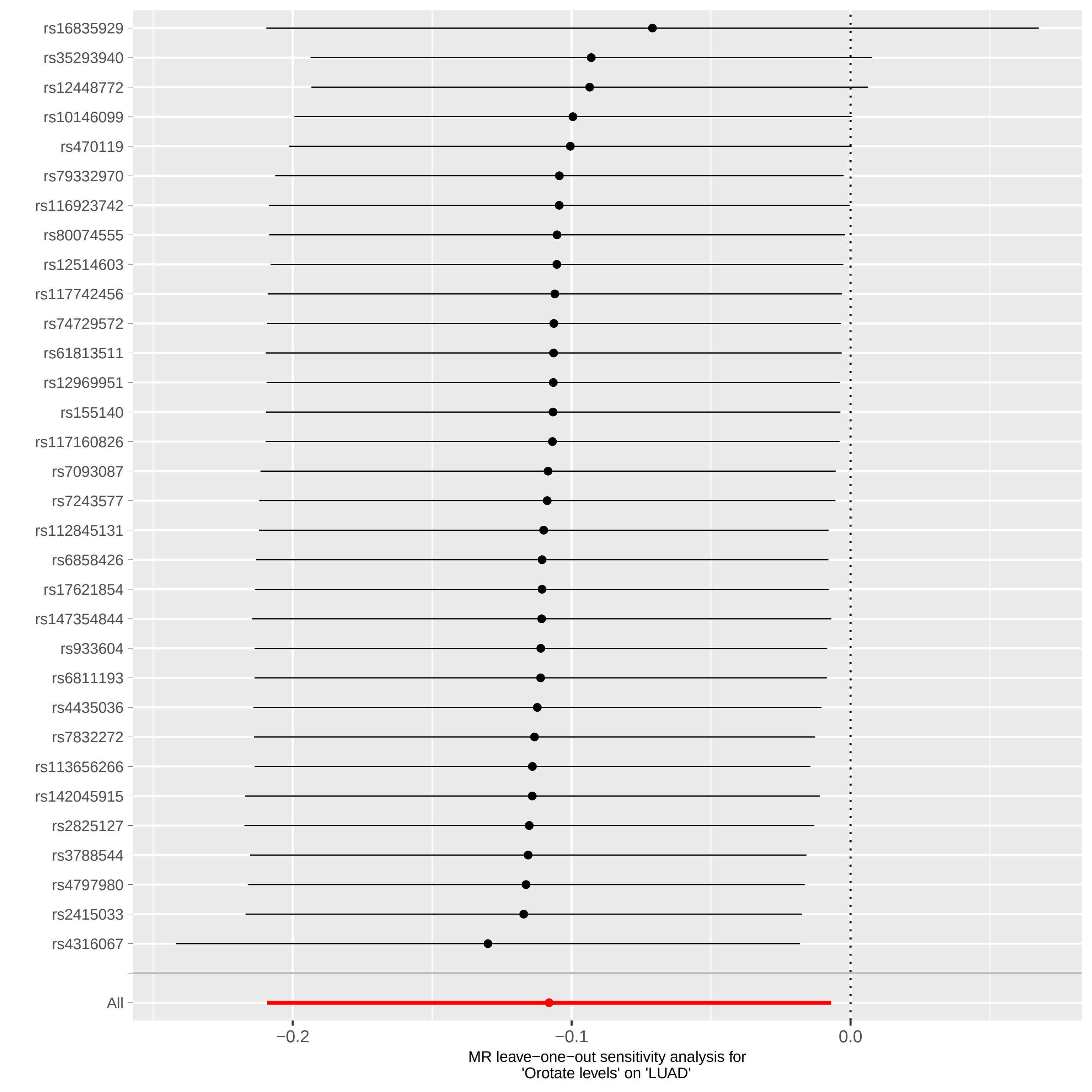

rs16835929
rs35293940
rs12448772
rs10146099
rs470119
rs79332970
rs116923742
rs80074555
rs12514603
rs117742456
rs74729572
rs61813511
rs12969951
rs155140
rs117160826
rs7093087
rs7243577
rs112845131
rs6858426
rs17621854
rs147354844
rs933604
rs6811193
rs4435036
rs7832272
rs113656266
rs142045915
rs2825127
rs3788544
rs4797980
rs2415033
rs4316067
All
−0.2
−0.1
0.0
MR leave−one−out sensitivity analysis for
'Orotate levels' on 'LUAD'
